# A comprehensive overview of focused ion beam-scanning electron microscopy (FIB-SEM) applications for the evaluation of outer retina

**DOI:** 10.3389/fcell.2025.1586029

**Published:** 2025-05-29

**Authors:** Sanjay Ch, Rayne R. Lim, Shermaine W. Y. Low, Deana G. Grant, Sam Patterson, Aparna Ramasubramanian, Ashish K. Gadicherla, Shyam S. Chaurasia

**Affiliations:** ^1^ Ocular Immunology and Angiogenesis Lab, Department of Ophthalmology and Visual Sciences, Medical College of Wisconsin, Milwaukee, WI, United States; ^2^ Electron Microscopy Core Facility, University of Missouri, Columbia, MO, United States; ^3^ Oxford Instruments Center for Advanced Microscopy- Electron Microscopy Core, Medical College of Wisconsin, Milwaukee, WI, United States; ^4^ Department of Cell Biology, Neurobiology and Anatomy, Medical College of Wisconsin, Milwaukee, WI, United States

**Keywords:** retinal degenerative diseases, SBF electron microscopy, FIB-SEM, electron microscopy, photoreceptors, retinal pigment epithelial cells

## Abstract

The retina is the light-sensitive inner layer of the eye, consisting of multiple cell types organized into ten distinct layers of neurons interconnected by synapses that play a crucial role in visual function. Any pathological alterations in this intricate structure can lead to vision impairment. Conventional electron microscopy techniques, including scanning electron microscopy (SEM) and transmission electron microscopy (TEM), provide our current understanding of ultrastructural defects in the retina. However, they are limited by their inability to image the complex three-dimensional (3D) structure layer-by-layer at a nanoscale resolution. Advanced electron microscopy techniques, including serial block face scanning (SBF), have emerged as a superior alternative to traditional imaging methods for enhancing the understanding of 3D segmentation at the nanoscale and revealing the ultrastructural architecture of the retina under both physiological and pathological conditions. This article provides a comprehensive overview of the advancements in SBF electron microscopy, emphasizing focused ion beam (FIB)-SEM for studying the interdigitation zone (IZ), which connects the cone outer segments to the retinal pigment epithelium (RPE) to enhance the understanding of retinal degenerative diseases such as inherited retinal disorders (IRDs), age-related macular degeneration (AMD), and diabetic retinopathy (DR). We have collated and discussed current literature alongside our recent work on FIB-SEM applications, particularly in examining the structural integrity of the outer retina. FIB-SEM can bridge the knowledge gap between structural insights and functional impairments through its state-of-the-art imaging and 3D segmentation capabilities. Additionally, it offers various applications for the pathological evaluation of retinal degenerative diseases.

## 1 Introduction

### 1.1 The retina and retinal pigment epithelium (RPE)

The retina resides in the posterior segment of the eye. It primarily processes visual information by converting light energy from photons into electric signals, which the brain processes to perceive real-time three-dimensional images of our vision (Masland, 2012; Hoon et al., 2014). The retina consists of multiple cell types, comprising intricate ten layers interconnected by neuronal synapses. The layers from the anterior to posterior are as follows: 1) Inner limiting membrane (ILM), 2) Nerve fiber layer (NFL), 3) Ganglion cell layer (GCL), 4) Inner plexiform layer (IPL) followed by 5) Inner nuclear layer (INL), 6) Outer plexiform layer (OPL), 7) Outer nuclear layer (ONL), 8) External limiting membrane (ELM), 9) Photoreceptor layer (PRL), and 10) retinal pigment epithelial (RPE) cells. The retinal cell types can be subdivided into three types: a) Photoreceptor cells- Rods and Cones; b) Neuronal cells–Amacrine, Horizontal, and Bipolar cells; c) Glial cells–Microglia, Astrocytes, and Muller cells (la Cour and Ehinger, 2005; Ptito et al., 2021).

RPE is a monolayer of pigmented microvilli cells that acts as a crucial barrier between the neural retina and the systemic circulation in the eye. The RPE is located on the basal surface of an extracellular matrix known as Bruch’s membrane, and on the other side, the apical microvilli interface with the outer segments of the photoreceptors. The tight junction between the cells serves as an outer blood-retinal barrier between the choroidal blood supply and the neural retina, facilitating the transport of essential nutrients and metabolites such as glucose, retinol, and fatty acids. RPE plays a significant role in the neural retina by improving spatial resolution, facilitating phagocytosis, and protecting the light sensitivity of the photoreceptors by recycling the visual pigments (Lakkaraju et al., 2020; Strauss, 2005).

### 1.2 Retinal degeneration involving RPE and photoreceptors

The retinal pigment epithelium (RPE) is a single layer of cells between the photoreceptors and the choroidal capillaries, contriving a crucial role in forming the outer blood-retinal barrier (oBRB). It facilitates the transport of nutrients and metabolites across the oBRB via Bruch’s membrane. It protects the retinal cells from photo-oxidative damage, which is critical for the structure and function of photoreceptor cells. The RPE also plays a decisive role in the visual cycle by converting all-trans-retinal to 11-cis-retinal and phagocytizing the shed outer segments of photoreceptors while producing essential factors that support retinal health (Zhang et al., 2023; Wang et al., 2024).

Photoreceptors are specialized neurons in the retina that convert light into electrical signals and send them to the brain for image processing. They are among the most copious and metabolically active cells in the body, with about 120 million in humans (Tonade and Kern, 2021; Lagnado and Baylor, 1992). Photoreceptor cells consist of two types-rods and cones. Rods are distributed throughout the retina and are responsible for low-light conditions or scotopic vision. In contrast, cones are densely populated towards the macula and highly concentrated in the fovea. These cones are responsible for color vision and high spatial acuity and are often active during daylight or photopic vision. The nocturnal rodent retina, such as that of mice or rats, is predominantly composed of rods, with a small percentage of about 3%–5% cones (Carter-Dawson and LaVail, 1979). Humans and primates have a fovea containing a circular area devoid of rods and tightly packed cones (Hendrickson, 1992).

**FIGURE 1:**
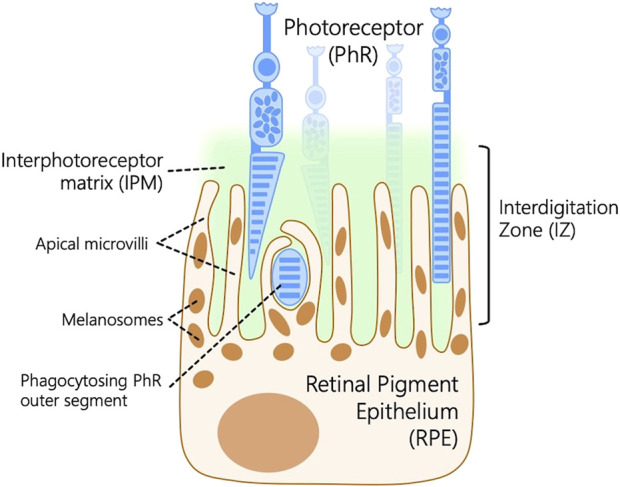
Schematic representation of the outer retina depicting the interdigitation zone (IZ), a retinal structure that connects the outer segments of photoreceptors (PhR) to the retinal pigment epithelium (RPE) microvilli embedded in the interphotoreceptor matrix (IPM) within the extracellular matrix located in the subretinal space between the PhR and the RPE.

The RPE works in tandem with the photoreceptors in the retina to maintain vision. Ocular diseases involving disruption of RPE-photoreceptor homeostasis account for common retinal diseases (Wang et al., 2024; Yang et al., 2021; Ishikawa et al., 2015), including diabetic retinopathy, retinitis pigmentosa, macular degeneration, Stargardt disease, cone-rod dystrophy, retinoschisis, and congenital stationary night blindness (Hoon et al., 2014). A lesser-studied component of the outer retina is the interdigitation zone (IZ), a retinal structure that connects the outer segments of photoreceptors to the RPE microvilli embedded in the interphotoreceptor matrix (IPM) within the extracellular matrix located in the subretinal space between the photoreceptors and the RPE (Figure 1). This matrix plays a crucial role in the survival and function of photoreceptors (Ishikawa et al., 2015). IPM is involved in key functional roles such as retinal adhesion to RPE, providing growth factors, delivering oxygen and other essential nutrients for the photoreceptors, and aiding in the transport of retinoids between photoreceptors and the RPE. Several genes responsible for encoding the proteins within the IPM have been linked to human-inherited retinal conditions, such as autosomal-recessive retinitis pigmentosa (Bandah-Rozenfeld et al., 2010), Sorsby’s fundus dystrophy (Weber et al., 1994), and Doyne honeycomb retinal dystrophy (Marmorstein et al., 2002).

Photoreceptors interact with the RPE via multiple signaling mechanisms, primarily engaging the subretinal space and the IPM (Lieffrig et al., 2023). Determining the ultrastructural architecture of individual photoreceptor cells and their interactions with their adjacent RPE cells provides valuable insights into ocular diseases, their signaling mechanisms, and the structural integrity of the outer retina (Ishikawa et al., 2015). This review outlines the preparation of retinal samples for state-of-the-art Focused Ion Beam-Scanning Electron Microscopy (FIB-SEM) technology, reviews previous research, and describes recent work from our lab on the outer retina to decipher the ultrastructural architecture of RPE and photoreceptors using FIB-SEM. This study may provide key insights into the structural components that enable retinal function to help develop new therapeutic strategies to combat photoreceptor loss or degenerative diseases in the retina.

## 2 Electron microscopy

The Electron Microscope, invented in 1931, transformed the microscopical examination of biological samples and revolutionized the understanding of the ultrastructural aspects of three-dimensional cells and tissues. This technology employs an accelerated electron beam as a light source for image formation. It can be broadly classified into Scanning Electron Microscopy (SEM) and Transmission Electron Microscopy (TEM) (Williams and Carter, 2009; Akhtar et al., 2018).

### 2.1 Scanning electron microscopy (SEM)

SEM is a powerful tool for examining the surface morphology, composition, and topography of a wide range of materials, biological and non-biological samples, with magnification up to 500,000 times using an electron beam for analysis. Electron beams are generated from sources, including tungsten filaments and Schottky field emission guns, and directed toward the specimen/sample. Non-conductive materials often require a metal coating with gold/platinum via sputter coating. When the primary electron beam interacts with the atoms on the surface of the sample, secondary particles are emitted from the point of interaction. These interactions help create an image that reveals the surface structure and characteristics of the material. SEM is widely used to depict cell surfaces, organisms, and their morphological evaluation. SEM can provide 3D projections of the sample, with acceleration voltages up to 30 kV and a theoretical resolution of 0.5–4 nm (Akhtar et al., 2018; Zhou et al., 2007; Postek, 1994).

### 2.2 Transmission electron microscopy (TEM)

TEM is extensively utilized to examine cellular structures by directing an electron beam through the thin sections of the sample to create image projections. It operates with a high-voltage electron beam generated by a heated cathode, directed through an aperture, and drawn into the magnetic lenses. The imaging process involves transmitting the electron beam through these thin sections, resulting in transmitted electrons being projected onto a fluorescence screen or a light-sensitive sensor for magnified imaging and observations. TEM enables visualization of the internal components of the cells, including protein structures, cytoskeletal filaments, and the arrangement of proteins in cell membranes. TEM can provide 2D projections of the sample, with the accelerated voltages ranging between 60 and 300 kV. TEM can magnify samples up to 1,000,000X with a 5 Å (angstroms) resolution. TEM requires thinner sample sections to be no thicker than 150 nm to ensure high-resolution images (Grover et al., 2022; Thompson et al., 2016; Williams and Carter, 2009).

## 3 Advances in electron microscopy

The traditional electron microscopy technique, particularly TEM, has been a primary tool for high-resolution ultrastructural studies of biological specimens; however, only 2D projections can be achieved through this technique. TEM has drawbacks, such as the requirement for thinner sections of the samples, as thicker sections result in multiple scattering events that compromise image quality. Consequently, an advanced 3D imaging technique is warranted to visualize the ultrastructure of the biological tissues with high resolution in thicker samples (Subramaniam, 2005; Narayan and Subramaniam, 2015). Recent developments in electron microscopy have enabled our understanding of the ultrastructure and function of intracellular organelles, compartments, and other cellular structures through 3D captured images at 1 nm resolution, covering depths of at least 1 μm, is made possible because of a few groups of techniques collectively termed volume electron microscopy (vEM) (Peddie et al., 2022). vEM combines methods based on SEM/TEM and produces a series of images by sequentially slicing the specimen into thin sections followed by imaging either sections or the surface. These images are later compiled to create a digital 3D representation of the specimen. vEM unveils the intricate structural details of organisms through the arrangement of membranes and organelles of cells and the architecture of tissues, which makes vEM an essential tool for understanding the biological complexity of life (Collinson et al., 2023). Currently, two main principles are employed to obtain 3D image data by electron microscopy. Firstly, it involves collecting serial sections, often described as serial section electron microscopy (ssEM), where either TEM or SEM can be used for imaging the serial sections on grids or tape. Secondly, it involves sequential SEM and TEM imaging on an exposed sample surface cut with a diamond knife (Serial Block Face, SBF) or focused ion beam (FIB). The material is repeatedly removed from the resin block, and the surface is imaged. In the former technique, sections are discarded, but the process is fully automated for efficient imaging (Collinson et al., 2023; Kizilyaprak et al., 2019b).

**FIGURE 2:**
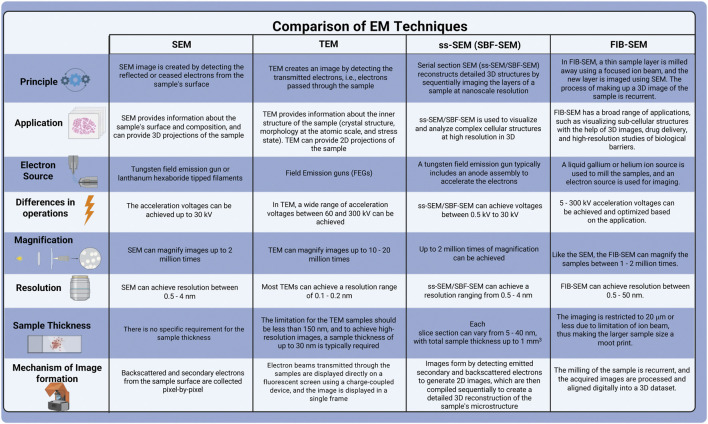
Comparison of various electron microscopy techniques.

The development of SBF electron microscopy addresses the limitations of serial sectioning. It achieves this by firmly securing the tissue onto an electron microscope stage, eliminating the risk of folding and debris recontaminating the section during collection. These advancements permit sequential imaging of the sample, avoiding compression issues and thus improving the image quality. SBF is uniquely defined by the cutting technique applied to resin-embedded samples, using either a diamond knife (SBF- SEM) or FIB-SEM. This review provides a concise overview of SBF- SEM and FIB-SEM techniques, comparing their respective advantages and disadvantages with traditional methods described in Figure 2, and elaborates on the application of FIB-SEM for characterizing the outer retina.

The introduction of electron tomography (ET) for imaging biological samples eliminated the limitations on sample properties. Previously, 3D EM techniques, such as single particle analysis and tissue 3D reconstruction of periodic structures, relied on structurally uniform or repetitive structures. However, biological molecules often exhibit natural variability, resulting in infrequent identical conformations. Furthermore, many large macromolecular assemblies remain uncrystallized despite recent advancements. ET overcomes these challenges by reconstructing unique, non-repetitive structures like cellular organelles. ET bridges the resolution gap between optical microscopy and high-resolution methods like single-particle analysis, making it ideal for correlative microscopy across atomic to cellular scales. The reconstruction of a 3D tomogram involves acquiring a tilt series of images taken at different angles, followed by image alignment, reconstruction, and visualization. These tilt series can be captured using various geometries, including single-, dual-, multiple-axis, or conical setups. The most common way of reconstructing the tomogram includes a weighted-back projection (WBP), algebraic reconstruction technique (ART), and simultaneous iterative reconstruction technique (SIRT) (Weyland and Midgley, 2004; Lemos et al., 2025; Bárcena and Koster, 2009).

Array tomography (AT) is an advanced imaging technique, particularly valuable for developmental and structural cell biology and related biomedical fields. It enables visualization of large tissue volumes, operating across a broad range of resolutions from ∼200 nm, as seen in conventional widefield light microscopy (LM), down to mere nm, comparable to standard EM. The term “array tomography” originates from the words: “array,” meaning a structured sequence, “tome,” meaning section, and “graphein,” meaning to record, reflecting the method’s core concept. In this approach, ribbons of ultrathin sections are positioned on a solid support and imaged by either LM or EM. By aligning these successive images, researchers can reconstruct three-dimensional (3D) representations of tissues. Unlike traditional ET, which builds 3D volumes from multiple 2D projections of a single ultrathin section typically less than 1 μm thick captured at varying tilt angles in a TEM, AT constructs 3D volumes by imaging a sequence of physical sections using a SEM. Reconstruction in ET relies on algorithms like weighted-back projection to transform the tilt series into 3D data. Nonetheless, the volume achievable for reconstruction is limited by TEM constraints, including section thickness limits (typically under 500 nm, depending on acceleration voltage). Creating comprehensive reconstructions of large cells or extensive tissue regions remains challenging (Wacker and Schroeder, 2013; Kolotuev, 2024) even by integrating multiple tomograms.

### 3.1 Serial block-face-surface scanning microscopy (SBF-SEM)

SBF-SEM is increasingly in demand for 3D imaging of the ultrastructure of resin-embedded biological samples over large areas. Biological samples stained with heavy metals are embedded in the hard resin, and the samples are sliced at a user-defined thickness (25–100 nm). A microtome is integrated into the microscope chamber. The sliced samples are typically imaged using an SEM that captures the scattered electrons from the sample’s surface. This process generates thousands of 2D stacked images, allowing for the analysis of ultrastructural details throughout the sample volume via 3D reconstruction (Narayan and Subramaniam, 2015; Denk and Horstmann, 2004). Previous studies employed conventional 2D electron microscopy techniques to analyze the retina, describing the ultrastructural characteristics of the RPE cells as they wrap apical microvilli around the outer segments of photoreceptors (Ferrington et al., 2016) and detailing the subcellular structures within the RPE cells (Altunay, 2007). The advent of SBF-SEM transformed 2D imaging in retinal pathology, enabling high-resolution, 3D reconstructions of retinal tissue at the ultrastructural level and revealing retinal degenerative phenotypes (Mustafi et al., 2014). A study performed on the mouse outer retina revealed the novel organization of the RPE using SBF-SEM (Ratnayaka and Keeling, 2022). The 3D data generated from imaging RPE cells provided cytoplasmic and nuclear volumes of RPE, the microvilli arrangement (length and angle) on the apical surface of the RPE, and the basolateral membrane spaces beneath the RPE. Therefore, 3D reconstruction of RPE cells demonstrated the interaction of photoreceptors and RPE at the subcellular level in the mouse retina (Keeling et al., 2020). In another study, SBF-SEM was employed to examine the ultrastructural organization of the basal labyrinth, which is essential for water and solute transport across the RPE under aging and disease conditions (Hayes et al., 2019). Their findings revealed that the organized structures of the basal labyrinth deteriorate with age and in choroideremia. The technique demonstrated how structural changes, influenced by osmotic variation, may regulate transport to maintain RPE volume, providing crucial insights into retinal pathology (Hayes et al., 2019). A study by Lindell et al. used SBF-SEM to visualize and map organelle distribution in 3D within apical processes of RPE and the cell body in photoreceptors (Lindell et al., 2023). SBF-SEM revealed spindle-shaped melanosomes in apical processes and a mixed population of electron-dense organelles in the apical half of the cell body. It also showed mitochondria mainly localized in the basal half, with a clear stratification of organelle zones and a circular arrangement near the nucleus, highlighting SBF-SEM’s effectiveness in revealing subcellular organization (Lindell et al., 2023).

SBF-SEM is an advanced high-throughput technique that has revolutionized the field of electron microscopy. However, it has certain limitations. The major drawback of SBF-SEM is that the sample is consumed after a single run, irrespective of the sample volume imaged, and samples are susceptible to charging artefacts. Additionally, the mechanical slicing of the sample with a diamond knife can introduce artefacts such as knife marks, folds, holes, stretching, or compression. The thickness of the slice can vary from sample to sample, potentially leading to inaccurate measurement of 3D-generated stacked images, and may result in poor-resolution images (Denk and Horstmann, 2004; Marshall et al., 2023; Titze and Genoud, 2016). These limitations associated with mechanical slicing using SBF-SEM can be addressed to a certain extent with the FIB-SEM, which mitigates compression and other artifacts by milling the sample at a preferred angle. The application and working principle of FIB-SEM are described in the subsequent section.

### 3.2 Focused ion beam-scanning electron microscopy

#### 3.2.1 Instrumentation

**FIGURE 3:**
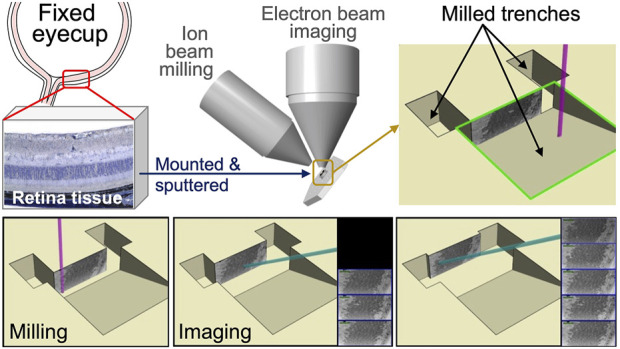
Schematic representation of mouse retinal sample preparation for FIB-SEM imaging. Mouse eyes were enucleated and fixed in electron microscopy fixative. The anterior chamber, including the cornea, ciliary body, and lens, was then dissected and removed. Posterior eye cups were submerged in HistoGel. Retinal samples were mounted and sputter coated onto a SEM stub using (COAT). Samples were then milled and imaged using FIB-SEM technology. Captured images were stacked to create the 3-D reconstruction of photoreceptor layers.

Modern SEMs integrate a focused ion beam employing a gallium source. The FIB columns are positioned at an angle of around 52°–55° relative to the electron column. The FIB and SEM columns have lenses, apertures, and electronic systems functioning independently. This setup facilitates both electrons and ion beams to interact with the sample simultaneously when positioned at the converging point, where beams converge typically at the working distance of 4–5 mm. FIB-SEM enables users to mill and image the sample at a specific location without disturbing the sample stage. The sample surface is perpendicularly tilted towards the ion beam. Then, with the help of FIB, a trench is milled into the sample region of interest to expose a polished cross-section for detailed imaging. The signals generated are detected by backscattered or secondary electron detectors, which are then used to create images. The schematic representation of mouse retinal sample preparation for FIB-SEM imaging is depicted in Figure 3. FIB-SEM setup enables efficient, stable milling and imaging of large volumes; however, it involves a minor compromise in resolution and signal quality because of the low beam currents and increased working distances (Heymann et al., 2009; Kizilyaprak et al., 2019a; Kizilyaprak et al., 2014).

#### 3.2.2 Sample preparation

**FIGURE 4:**
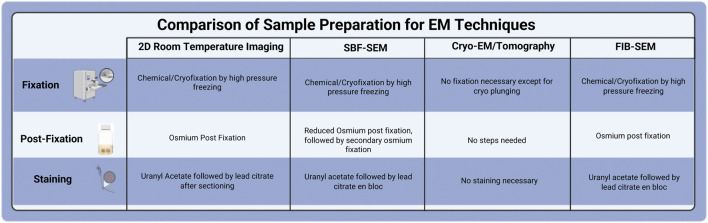
Comparison of Sample Preparation for various EM techniques.

Sample preparation plays a crucial role in the success of electron microscopy for biological samples. The specimen must endure challenges such as the vacuum environment of the EM, as well as the high energy and current of the electron or ion beams. The sample should possess the structural stability for volume slicing and provide adequate image contrast. The protocols are often adjusted to meet the specific characteristics of the sample, the target structure, and imaging methods. The technical characteristics of FIB-SEM and SBF impose additional barriers to adequate sample processing, including lower signal generation and an increased charging effect compared to traditional thin-section TEM. SEM-based imaging methods rely on backscattered electrons as the primary signal for image generation, resulting in lower contrast than the transmission signal employed by TEM. Furthermore, surface charging presents a significant barrier to quality SEM imaging, as electrons accumulate on the surface of any biological tissue that is not adequately grounded. Additional heavy metal staining steps can mitigate these effects by ensuring adequate conductivity and increased contrast required by SEM-based techniques. The resin of choice is also altered to provide sufficient stability under the ion beam during the milling procedure. These necessary processing alterations combine to make FIB-SEM imaging of biological tissue a reliable, albeit more in-depth, methodology than sample processing for TEM. Below is a general overview of the critical steps in sample preparation before imaging, such as prefixation, sample size optimization, chemical fixation, and other imaging procedures (Drobne, 2013; Titze and Genoud, 2016; Xu et al., 2017; Courson et al., 2021). The comparison of sample preparation among various EM techniques is summarized in Figure 4.

##### 3.2.2.1 Prefixation

The tissue collected from the animal is pre-fixed with a mixture of aldehydes, typically 2%–4% paraformaldehyde and 2%–4% glutaraldehyde, followed by dehydration of the sample and embedding in epoxy resin (Pang and Xu, 2023). Alternatively, Cryo-fixation through high-pressure freezing, followed by freeze substitution to transition the sample into resin, effectively captures rapid subcellular events within milliseconds while minimizing the shrinkage artifacts. Chemical fixation and cryo-fixation can be chosen depending on the sample size. High-pressure freezing is suitable for samples around 200 μm thick, whereas larger samples, measuring in mm, can be preserved using chemical fixation (McDonald, 2009; Peddie et al., 2022; McDonald, 2014).

##### 3.2.2.2 Chemical fixation

Various tissues, such as liver, muscle, retina, and cell culture samples, are initially fixed with a 2%–4% aldehyde mixture (glutaraldehyde and formaldehyde) in cacodylate buffer. This is followed by a 2 h treatment with equal parts of 2%–4% osmium tetroxide and 1%–3% potassium ferricyanide in a buffer solution at a pH of 7.2 (Tapia et al., 2012; Hua et al., 2015; Lu et al., 2022). To reduce the charging effects, the image contrast of the FIB-SEM is improved by staining the samples with *en bloc* staining. An additional step with thiocarbohydrazide can be included in enhancing the osmium uptake of the sample, as thiocarbohydrazide binds with the osmium tetroxide (Kurzer and Wilkinson, 1970). Additional *en bloc* staining utilizes 2% aqueous uranyl acetate to add contrast and serve as a tertiary fixative. After a few hours of incubation with uranyl acetate, the samples are dehydrated using ethanol or acetone gradient and embedded in epoxy resin (Tapia et al., 2012; Ronchi et al., 2021).

##### 3.2.2.3 Dehydration and resin embedding of the samples

After the chemical fixation, to safeguard the samples from the vacuum environment of the electron microscope and to enable ultra-thin sectioning, the water inside the cells and tissues is removed through dehydration using a solvent compatible with later resin infiltration. Chemically fixed samples are dehydrated through a graded series of ethanol or acetone, whereas the high-pressure frozen samples undergo freeze substitution (McDonald, 2014; Hoffman et al., 2020).

##### 3.2.2.4 Milling

*In situ*, the microtome is replaced with milling in advanced vEMs such as FIB- SEM. The milling conditions, such as ion-beam acceleration voltage and current, vary depending on the sample surface’s rigidity. It provides the unique advantage of achieving thinner slices at about 5 nm with FIB compared to SBF (around 25 nm) and provides better resolution with a high signal-to-noise ratio (Xu et al., 2017). Since the direction of ion beam milling is perpendicular to other sectioning methods, it is crucial to account for this during the experimental design to ensure that the target region remains accessible to the electron beam. However, as the ion beam continues to mill the block surface, it gradually loses energy, which limits the precision of deeper cuts. To enhance speed and throughput, samples have been imaged concurrently across multiple FIB-SEM instruments, which are more stable and less prone to errors during prolonged imaging runs (Xu et al., 2017; Baena et al., 2021).

#### 3.2.3 Image processing for 3D reconstruction

Two-dimensional observations provide limited insights, making capturing geometry-sensitive features challenging and comparing them to three-dimensional data thoroughly. The FIB-SEM 3D imaging directly visualizes the sample surface at nano-scale resolution through the sequential 2D image sets into a detailed 3D model (Nan and Wang, 2019). SEM converts signals from secondary and backscattered electrons into digital images that reveal the sample’s morphology and crystallography with contrast based on topography. FIB-SEM enhances this by collecting extensive datasets from biological tissues, enabling the automatic acquisition of thousands of 2D images. These images can be reconstructed into 3D models, making ultrastructural studies more efficient.

FIB-SEM data processing comprises four steps: 1) stack alignment, 2) removal of FIB artifacts, 3) image optimization, and 4) segmentation. Stack alignment is the first step in data processing; it is a sensitive procedure that requires sub-pixel accuracy and stacking of the collected SEM images (Prill et al., 2013). Some algorithms used for stacking include the StackReg and TurboReg plugins, followed by stack alignment and removal of artifacts to address the “curtaining effect” caused by FIB milling. Fast Fourier transform (FFT) eliminates the artifacts, using a zero-filter mask shaped as a vertical rectangle and aligned along the central vertical axis to remove unwanted noise or distortions. An alternative method involves an algorithm designed to recover clean images affected by two main distortions: stripe artifacts resulting from variable milling rates and layered patterns caused by partial material removal during the milling process. Image optimization takes place after artefact removal, to enhance the feature localization and differentiation during segmentation, which requires adjusting contrast and brightness on individual images. Commonly used techniques include the Gaussian 3D filter and background equalization methods like “Gradient Xterminator” to minimize noise (Mura et al., 2023). The final step of the data processing is the segmentation, which can be categorized into three main types: Manual, semi-automatic, and automatic (Terao et al., 2017). Manual segmentation is a labor-intensive process requiring a detailed image inspection and delivering a highly precise outcome. The semi- and fully automatic segmentation procedures utilize the global or local thresholding techniques applied to grayscale values to distinguish features within an image (Farhana Faisal et al., 2019). Automatic segmentation utilizes a commonly known watershed algorithm, which interprets the darker (low grayscale) and lighter (high grayscale) areas of the image, allowing effective segmentation. On the other hand, semi-automatic segmentation optimizes the threshold values in the first place, followed by a detailed manual check to achieve highly effective segmentation (Mura et al., 2023).

#### 3.2.4 Application of FIB-SEM in deciphering retinal structure

Understanding changes in retinal network topologies during disease progression is crucial for evaluating treatment and management strategies for vision-threatening retinal disorders. Expanding retinal connectomics into retinal pathoconnectomics provides insights into how degenerative retinal diseases disrupt these networks, highlighting where failures can lead to vision loss (Pfeiffer et al., 2019). Advanced electron microscopy techniques such as FIB-SEM play a vital role by enabling high-resolution 3D imaging of retinal structures. FIB-SEM allows researchers to visualize ultrastructural and subcellular changes in retinal cells. These advanced techniques provide essential information about cellular architecture and changes in neuronal synaptic networks in degenerative diseases. This detailed imaging helps identify key failure points in the retinal network, aiding in diagnosing and developing targeted therapies for vision restoration (Sigulinsky et al., 2024).

**FIGURE 5:**
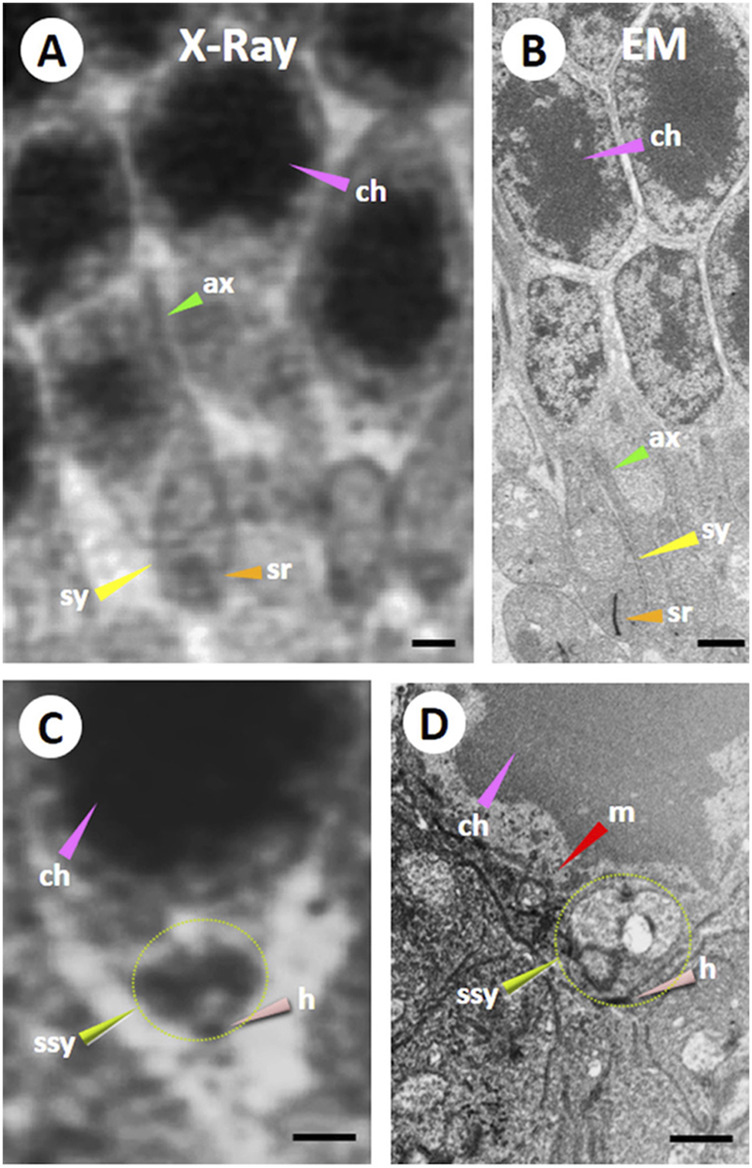
Comparison of Ptychographic hard X-ray computed tomography (PXCT) and Focused Ion Beam-Scanning Electron Microscopy (FIB-SEM) imaging in the mouse outer retina. A resin-embedded retina was imaged using PXCT **(A,C)** and FIB-SEM **(B,D)** in a similar region of the outer plexiform and outer nuclear layers (OPL and ONL) from a wild-type mouse retina. Both images revealed nuclei packed with dense chromatin (ch; pink arrowheads), axonal fibers (ax; green arrowheads), synaptic termini (sy, yellow arrowheads), and synaptic ribbon (sr; orange arrowheads). Certain neural cell bodies exhibited high-resolution 3D features surrounding chromatin in FIB-SEM images, including several somatic synapses (ssy; lime green arrowhead and dotted line circle) observed in the large PXCT measurement **(C)**. Also, the somatic synapse (ssy; lime green arrowhead and dotted line circle) on its hilus (h; salmon arrowhead) was observed near a large mitochondrion (m; red arrowhead) in **(D)**. Scale bar 1 µM. Reprinted with permission from Panneels et al. (2021) 134, jcs258561. doi:10.1242/jcs.258561.

A previous report used ptychographic hard X-ray computed tomography (PXCT) and FIB-SEM techniques to compare images taken from the retinal cellular and sub-cellular structures in the outer plexiform layer (OPL) of the VPP mouse (an autosomal-dominant retinitis pigmentosa (RP) animal model) and wild-type mouse to investigate the feasibility of techniques for analyzing the specimen with retinal degeneration (Panneels et al., 2021). The images from the RP mouse (Figure 5) revealed that significant tissue degeneration was evident compared to the wild-type mouse at the same age. The RP-mouse OPL layer no longer exhibits the rod synaptic terminals (sy), known as spherules or the axonal fibers (ax) (Figure 5D). The 3D volume of the OPL reveals widespread fibrosis, along with bipolar cell bodies, vascular structures, and remnants of synapses (Panneels et al., 2021).

**FIGURE 6:**
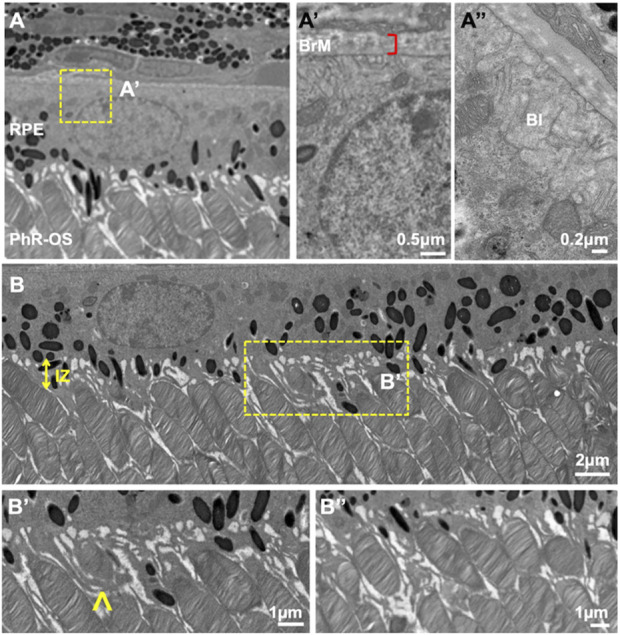
TEM images of wild-type C57B/1/6J mouse retina. TEM images of the outer retina were captured using a JEOL JEM 1400 microscope at 80 kV on a Gatan Ultrascan 1000 CCD at 1200X magnification **(A)**. Images were further enlarged to analyze the mice’s retinal layers. The RPE was polarized with apical microvilli and BI **(A″)** adjacent to the BM **(A′)**. Electron-dense pigment granules are distributed in the middle and apical portions of the RPE, with some located in the apical processes. IZ is uniformly spaced throughout the Retina **(B)**. PhR-OS were regularly spaced across the RPE, and apical processes were seen interdigitating with individual PhR-OS **(B, B″)**. Microvilli engulf PhR discs in preparation for phagocytosis and enter them into the RPE **(B′)**, arrow. TEM, Transmission Electron Microscopy; RPE, Retinal Pigmented Epithelium; BI, basal infoldings; BM, Bruch’s Membrane; IZ, Interdigitation zone; PhR-OS, Photoreceptor Outer Segment.

In our previous work on the outer retina (Lim et al., 2018), TEM and FIB-SEM were employed to evaluate the ultrastructural architecture of the RPE-photoreceptor complex (Figure 6). The workflow TEM images revealed the ultrastructure of retinal layers, such as Bruch’s membrane (BM) and the interdigitation zone (IZ). The outer retina showcased individual photoreceptors, the linkage between their inner and outer segments through photoreceptor cilia, and their interaction with RPE ciliary processes. The RPE was polarized, with apical microvilli oriented towards the photoreceptors and basal ends on the basal side next to the BM. The arrangement of BM, RPE mitochondria, photoreceptors, and pigment granules analyzed in this study using TEM was consistent with the previous observations in the human RPE (Lindell et al., 2023).

**FIGURE 7:**
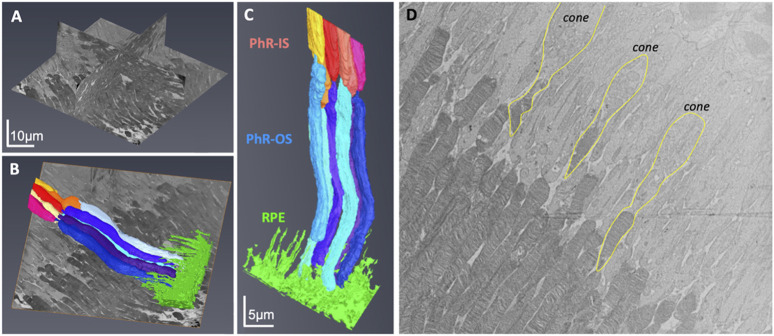
Focused Ion Beam-Scanning Electron Microscopy (FIB-SEM) imaging of Photoreceptors (PhR) in mouse retina. Orthogonal view of the milled block along the different axes **(A)**. Individual rods and the RPE were 3-D reconstructed and digitally pseudocolored **(B,C)**. Most photoreceptors were rods, with the cones (outlined in yellow) easily identified by a wider PhR-IS and accumulation of mitochondria in the center of the IS **(D)**. PhR-IS, Photoreceptor Inner Segment; PhR-OS, Photoreceptor Outer Segment; RPE, Retinal Pigmented Epithelium.

**FIGURE 8:**
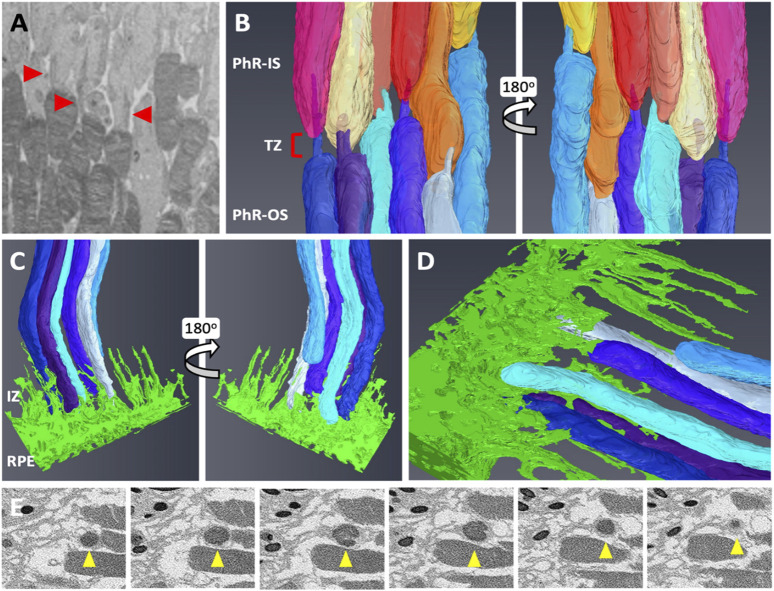
Focused Ion Beam-Scanning Electron Microscopy (FIB-SEM) segmentation revealed an interconnecting cilium and an interdigitation zone in the RPE-photoreceptor complex. The cilium in rods (red arrowhead) was identified and digitally segmented as part of the PhR-OS **(A,B)**. Digital reconstruction revealed the cilium and TZ in every rod **(B)**. At the RPE-photoreceptor complex, RPE apical processes extend upwards to the PhR-OS distal tips, forming the IZ **(C,D)**. Sequential image slices taken at the distal tip demonstrate the engulfment of a shed disc (yellow arrowhead) by the RPE microvilli **(E)**. RPE, Retinal Pigmented Epithelium; PhR-OS, Photoreceptor Outer Segment; PhR-IS, Photoreceptor Inner Segment; TZ, Transition Zone; IZ, interdigitation Zone.

Next, the retinal tissue was processed using FIB-SEM to examine the photoreceptors connecting the cilia and interdigitation zone (IZ) of the photoreceptor outer segments with RPE microvilli. The individual rod inner and outer segments and RPE were pseudocolored to identify the ciliary interactions and visualize the IZ (Figure 7). The rod outer segment (ROS) volume was slightly larger than the dimensions reported previously (Carter-Dawson and LaVail, 1979). Additionally, the segmentation of the photoreceptor complex revealed the interconnecting cilium and IZ. The RPE (green) was rich in apical microvilli that extended into the photoreceptor space. Sequential image slices of IZ revealed the encapsulation of a shed disc by the RPE microvilli, which would eventually be phagocytosed by the RPE (Figure 8).

**FIGURE 9:**
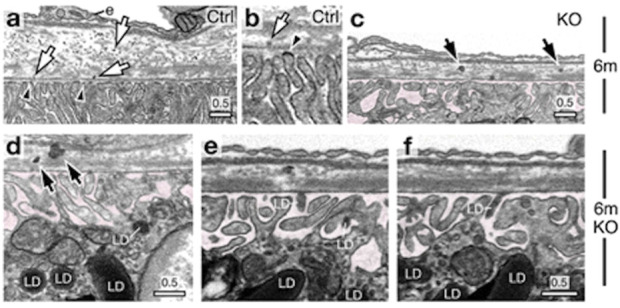
Focused Ion Beam-Scanning Electron Microscopy (FIB-SEM) images of a 6-month-old wild-type mouse RPE-Bruch’s membrane (BM)-choroid complex show lipoprotein-like particles distributed through BM (indicated with arrows), emerging from dark lipid-raft regions of the basal plasma membranes (arrowheads) **(a,b)**. FIB-SEM images of 6-month-old knockout (KO) mice display spherical LD-like structures within BM, accompanied by several lipoprotein-like granules arrows in **(c,d)**. These BM-localized LDs resemble those found in RPE basal cells **(d)** and within the sub-RPE space **(e,f)**highlighted in pink. Reprinted with permission from Chuang et al. (2022) 13:374. doi:10.1038/s41467-021-27935-9.

FIB-SEM is a powerful tool for precisely examining RPE-photoreceptor complex abnormalities. Our data revealed changes in the ultrastructure of the IZ, ciliary connections, and RPE polarization, which may allow early detection of structural abnormalities, such as defective disc shedding, impaired cilia, or altered thickness in retinal layers (Low et al., 2023). These insights enhance our understanding of the progression of retinal degenerative diseases. FIB-SEM tomograms were biocomputed to study the characteristic feature of mouse rod photoreceptor nuclei, the proportion of heterochromatin, and the number, density, and distribution of nuclear pore complexes (Hoang et al., 2017). In a recent study (Chuang et al., 2022), the authors examined an RPE-specific chloride intracellular channel 4 (CLIC4) protein knockout mouse model, demonstrating clinical and histological hallmarks of dry age-related macular degeneration (AMD). Since the mechanism behind drusen-like deposit formation in the RPE is not yet understood, the authors imaged the RPE-BM region using 3D electron microscopy, revealing previously unrecognized export of lipid droplets from the dark lipid-raft areas of the RPE basal plasma membranes into the BM (Figure 9). This led to the hypothesis that these lipid droplets bind to the lipoproteins, leading to retention in BM and impaired choroidal clearance, thereby forming drusen-like basal linear deposits. Their data further linked lipid membranes to drusen formation, paving the way for new avenues for the management of AMD. FIB-SEM played a crucial role by providing high-resolution images revealing the previously unrecognized export of lipid droplets from the RPE into BM. Thus, FIB-SEM helped establish the connection between RPE lipid dysregulation and drusen formation, offering new insights into the mechanisms underlying AMD pathogenesis (Chuang et al., 2022).

**FIGURE 10:**
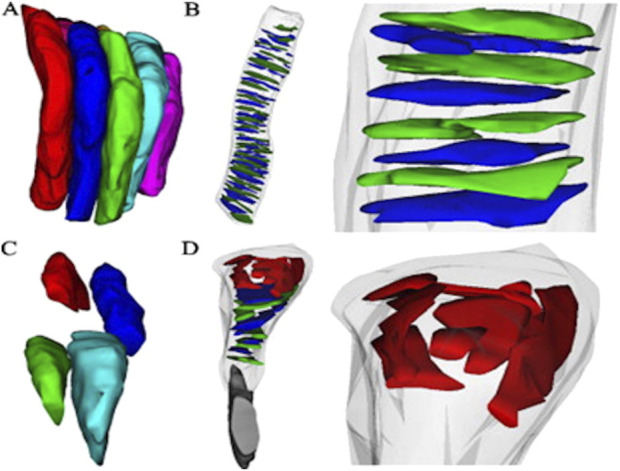
Three-dimensional reconstructions from Serial Ion Ablation (SIA)-SEM data provide insights into the packing structure, internal components of photoreceptors, and disease-related alterations **(A)** Reconstructed rod photoreceptors from a wild-type (WT) mouse retina, with an average diameter of 1.2 ± 0.1 µm, are shown to align closely and pack compactly. Internal elements of photoreceptors in WT rods reveal **(B)** orderly stacked discs, with an average disc diameter of 1.1 ± 0.3 µm (n = 41 discs) **(C)** In the *Nrl* ^*−/−*^ retina, photoreceptor packing density significantly decreases due to irregularly shaped outer segments, averaging 1.0 ± 0.2 µm in diameter. Additionally **(D)** *Nrl* ^*−/−*^ photoreceptors exhibit atypical structures at the retinal pigment epithelium (RPE) interface, with sizes ranging from 0.4 to 0.8 µm. Reprinted with permission from Mustafi et al. (2011) 198:70–76. doi:10.1016/j.jneumeth.2011.03.013.

In another study, Mustafi et al. (2011) used the FIB-SEM to visualize rod photoreceptors and their internal disc structures in wild-type (WT) mice (Mustafi et al., 2011). Since the mouse retina predominantly comprises rod photoreceptors, the authors fine-tuned magnification and ion beam parameters to explore rod arrangements/packing using a trench exposed to the area of interest, allowing visualization of photoreceptor architecture in stacks, thus resulting in 3D reconstruction (Figure 10). Similarly, the authors applied FIB-SEM to a diseased mouse model lacking the neural retina leucine zipper transcription factor (*Nrl* ^*−/−*^), the photoreceptors displayed bulbous outer segments with disrupted packing. Ion-beam milling and 3D reconstruction further revealed disorganization in the internal structure of the retinal outer segments (Mustafi et al., 2011).

## 4 Current challenges and future directions

**FIGURE 11:**
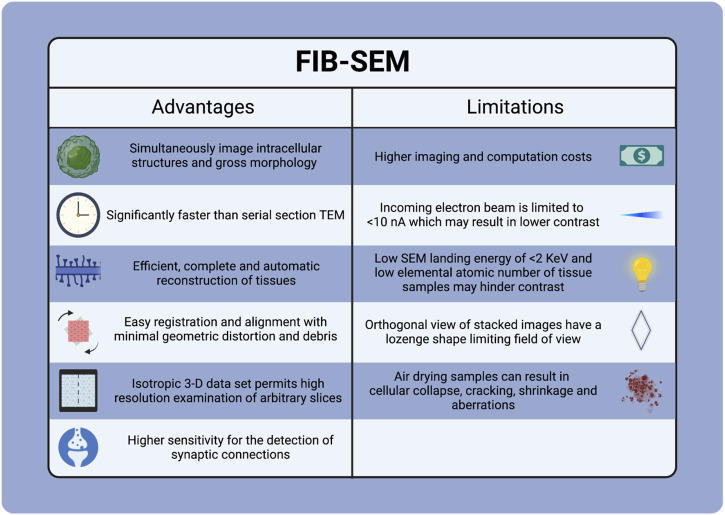
Advantages and Limitations of FIB-SEM Tecnique.

FIB-SEM is in the early phases of development and encounters several challenges (Figure 11). These include the high costs associated with instrumentation, facilities, maintenance, operating expenses, and training. Additionally, it has longer acquisition times, artifacts from cross-sectioning, shadowing effects, limits on re-imaging due to sample consumption during image milling and acquisition, and a requirement for substantial digital storage to enable efficient data transfer and processing for volumetric imaging applications. There are also considerable commitments required for learning and training personnel to operate the instrumentation in the facility.

Future advancements in FIB-SEM will enhance our understanding of different retinal diseases, which pose microscopic challenges beyond the capabilities of conventional imaging methods, such as SEM and TEM. The structural damage associated with retinal pathological conditions can be visualized and analyzed through 3D architectural reconstruction of retinal layers, potentially offering valuable insights into the spatial relationship between cells, layers, and structures at the nanoscale resolution. These recent technical advancements would aid in understanding retinal diseases such as AMD and glaucoma, which gradually affect various retinal layers over time.

Ultrastructural and subcellular imaging of cellular components such as synapses, mitochondria, and endoplasmic reticulum can provide valuable insights for studying the mitochondrial anomaly in retinal cells. In conditions such as retinitis pigmentosa, imaging the ultrastructural details of the synaptic connections in photoreceptors offers critical perspectives into understanding the functional correlation with vision loss. The applications of FIB-SEM can be examined in various ways, offering significant potential for enhancing our understanding of disease pathology, facilitating early diagnosis, and advancing the future of personalized treatment for retinal diseases. FIB-SEM can also work synergistically by combining advanced artificial intelligence (AI) algorithms to analyze large retinal disease datasets for accurate and faster diagnoses. Moreover, it can be integrated with conventional optical coherence tomography (OCT) imaging techniques, providing multi-scale imaging in both micro and nano projections of the retinal images. This multi-modality approach would significantly enhance the early detection of several retinal diseases (Saleh et al., 2022; Sorrentino et al., 2024).

## 5 Conclusion

Advancements in electron microscopy have revolutionized the imaging of biological tissue at high resolutions in nanoscale magnifications. FIB-SEM offers unique advantages for layer-by-layer segmentation followed by 3D image reconstruction, providing researchers with a better structural understanding of the retinal tissue. In this review, we collated and discussed the intricate subcellular structures of the outer retina, especially the complex interactions of RPE-photoreceptors and structural alterations of the retina in pathological conditions. In conclusion, the full potential of FIB-SEM could be explored further in various retinal pathological conditions, helping researchers understand the morphological changes and functional outcomes during both the developmental phases and disease states of the retina.
